# Active Safety Surveillance Using Real‐World Evidence (ASSURE) Implementation: Transparent and Reproducible Real‐World Evidence Standardized Framework to Support Safety Signal Evaluation

**DOI:** 10.1002/pds.70435

**Published:** 2026-07-24

**Authors:** Kevin Haynes, Jenna Reps, Jill Hardin, Eva‐Maria Didden, James Gilbert, Joel Swerdel, Justin Bohn, Mitchell M. Conover, Amir Sarayani, Anthony G. Sena, Emily Yost, Valerie van Baalen, Kourtney Davis, Patrick Ryan, Martijn Schuemie

**Affiliations:** ^1^ Johnson & Johnson Horsham Pennsylvania USA; ^2^ Johnson & Johnson Raritan New Jersey USA; ^3^ Johnson & Johnson Allschwil Switzerland

**Keywords:** pharmacovigilance, real world evidence, safety surveillance

## Abstract

**Purpose:**

To summarize experience with Johnson & Johnson's novel Active Safety Surveillance Using Real‐world Evidence (ASSURE) program for producing efficient, transparent, applicable, and impactful RWE to support routine pharmacovigilance processes.

**Methods:**

ASSURE follows a stepwise framework that encompasses database diagnostics to assess fit‐for‐purpose real‐world data (RWD) sources in the US, France, Germany, Australia, and Japan, phenotype development, query specification, and analytic implementation, objective study validity diagnostics, and standardized reporting. Licensed RWD sources are transformed into the Observational Medical Outcomes Partnership (OMOP) Common Data Model (CDM), from which we generate evidence for safety signal evaluation questions using a suite of open‐source analytic tools made publicly available through the OHDSI community. For each signal evaluation request involving a medication exposure and outcome(s), we conducted population characterization and population‐level causal effects estimation. Target medication, comparator medication, and indication cohorts utilize predefined phenotypes, when available, for rapid analytics. We provide an evaluation of the initial implementation of this framework, including results from database diagnostics, phenotype development, and objective study validity diagnostics applied prior to unblinding evidence to prevent exposing biased estimates. We defined efficiency as the time from query to results and integration of results into safety decisions.

**Results:**

The initial 19 months of the ASSURE program supported 110 safety signal evaluations across 24 unique products that arose from 47 requests. 74 evaluations (67%) passed study diagnostics and yielded effect estimation results with 62 (54%) providing propensity‐score adjusted comparative cohort method results and 40 (36%) providing self‐controlled case series results. Many analyses failed one or more diagnostics, preventing the generation and unblinding of potentially biased results: database (7 evaluations), phenotype specification (3 evaluations), or the requisite objective study validity diagnostics (26 evaluations). These 26 evaluations (24%) all provided population characterizations of exposure, indication, and outcome, including incidence rates.

**Conclusions:**

The ASSURE framework and implementation process, in collaboration with safety management teams, enables rapid response to medical product safety signals under evaluation and produces actionable RWE that supports pharmacovigilance decision making within regulatory timeframes.

## Key Points

- We describe an approach to efficiently conduct timely RWD‐based pharmacovigilance medical product surveillance analyses utilizing a network of RWD data sources transformed to the OMOP CDM with application of standard analysis modules for characterization and causal effect estimation.
- Fundamental components of the ASSURE framework are the uniform application of open community data standards across disparate data and reproducible use of open source standardized analytic tools to facilitate the efficient design and implementation of analyses, and consistent interpretation of results.
- Key steps in data processing include transforming insurance claims and EHR RWD into the OMOP CDM, and rigorous quality checks to ensure data completeness, validity, and consistency.
- Key steps in generating RWE for signal evaluation involve translation of a safety concern into the standardized ASSURE framework, evaluation of objective study validity diagnostics which prevent the unblinding of potentially biased results that fail to meet prespecified objective criteria, and reporting results to inform regulatory decisions.

## Plain Language Summary

- What ASSURE is
  ○ ASSURE (Active Safety Surveillance Using Real‐world Evidence) is a program developed to quickly and reliably check whether medicines are safe using real‐world health data—like insurance claims and electronic health records—from multiple countries (US, France, Germany, Australia, and Japan).
- How it works
  ○ We convert different health data sources into a single, common format so they can be analyzed the same way.
  ○ We use publicly available tools and clear clinical definitions for who is exposed to a medicine, who is not, and what counts as the health outcome of concern.
  ○ Before looking at the final results, we run a set of quality checks to make sure the data and methods answer the question correctly.
  ○ If the checks pass, we report whether the medicine appears to affect the risk of the outcome. If checks fail, we stop and share what we can (e.g., how often the medicine or outcome occurs) so decisions are not based on unreliable analyses.
- Key findings from the first 19 months.
  ○ ASSURE handled 110 safety analyses for 24 different products from 47 requests.
  ○ 67% (74 analyses) passed the quality checks and produced effect estimates.
  ○ 54% (62 analyses) produced adjusted comparative results (comparing users of the medicine to an appropriate comparison group).
  ○ 36% (40 analyses) produced self‐controlled results (which compare a person while taking or not taking medicine).
  ○ 24% (26 analyses) failed one or more important checks and therefore did not produce reliable comparison; for those, we still provided basic counts and rates.
- Why these matter
  ○ The approach makes safety reviews faster, more transparent, and more reliable by using the same standards and reusable tools across many patient populations in different data sources.
  ○ Running thorough checks before releasing results helps avoid mistaken or biased conclusions and supports better and timely decisions about medicine safety.

## 1 Introduction

Real‐world data (RWD) captured during routine clinical care, such as health insurance claims and electronic health records (EHRs), are widely used for pharmacoepidemiology research and real‐world evidence (RWE) generation [1, 2]. Regulatory agencies have established platforms that utilize large healthcare databases for post‐marketing medical product safety surveillance, e.g., FDA's Sentinel System [3], EMA's Data Analysis and Real‐World Interrogation Network (DARWIN EU) [4], Health Canada's Canadian Network for Observational Drug Effect Studies [5] (CNODES). Modelling off these foundational safety surveillance systems, we (Johnson & Johnson) have developed the Active Safety Surveillance Using Real‐world Evidence (ASSURE) framework to efficiently produce transparent, robust, and reproducible, RWE to support pharmacovigilance (PV) safety signal evaluation activities. ASSURE facilitates rapid turnaround even for complex study designs and provides evidence that can impact time‐sensitive decisions, while conforming to current best practices for analyzing observational healthcare data. Database and study diagnostics are utilized to minimize bias and ensure only reliable results are unblinded for interpretation.

The ASSURE framework follows guidance put forth by the Joint Task Force between the International Society for Pharmacoeconomics and Outcomes Research (ISPOR) and the International Society for Pharmacoepidemiology (ISPE), which seeks to advance the transparency and reproducibility of operational study parameters used to create analytic datasets and analyses from longitudinal healthcare databases [6]. Safety signal evaluations aim to provide regulatory responses within 2–3 months and, therefore, require an efficient system to provide a rapid response. Safety signals can come from regulatory authorities, literature reviews, or spontaneous reports. Pharmaceutical companies are required to evaluate safety signals to inform labeling decisions and health authority requirements. Meeting these timelines is challenging with conventional approaches to RWE generation, given the need to define and evaluate computable phenotypes, develop and test programming code, and execute the analyses across available data sets in a network. The ASSURE framework is based on a set of standardized inputs and execution of standardized analytics on standardized RWD resources available within Johnson & Johnson to efficiently produce standardized results to support signal evaluation decisions. While these RWD and analytic standards follow the Observational Health Data Sciences and Informatics (OHDSI) practices, they may still be subject to potential bias inherent to observational data [7]. The ASSURE framework incorporates a set of objective study validity diagnostics [8] to prevent the output of potentially biased results. This blinding of potentially biased results prevents potentially biased estimates from being reviewed and potentially misinterpreted [8]. This manuscript describes the ASSURE framework and characterizes its efficiency and impact of the results on signal evaluation decision making over the first 19 months of implementation.

## 2 Methods

### 2.1 Data Preparation

Johnson & Johnson had access to licensed RWD sources, including administrative claims and EHRs from the US, France, Germany, Australia, and Japan. A full description of the databases is beyond the scope of this manuscript. Briefly, the US claims data sources contained administrative billing details from outpatient, inpatient, and pharmacy services, EHR resources contained outpatient encounters primarily from primary care. Ten data sources from multiple countries (5 US administrative claims, 1 US EHR, 1 France EHR, 1 Germany EHR, 1 Australia EHR, and 1 Japan EHR) were mapped to the Observational Medical Outcomes Partnership (OMOP) Common Data Model (CDM) v5.4 [9]. Standardizing the data into a common format makes it possible to deploy the same analytical programs and software across different data sources. The mapped data were characterized and evaluated for quality. The OHDSI Data Quality Dashboard was used to perform over 3000 data quality checks [10, 11]. Data were only used in the ASSURE framework if they were determined to be of general satisfactory research quality prior to evaluating fitness‐for‐use. All databases are de‐identified and determined to be except from IRB review.

### 2.2 Framework

**FIGURE 1:**
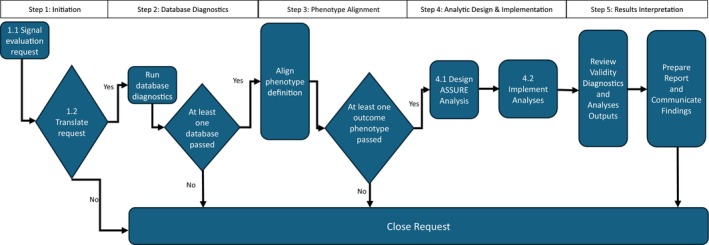
ASSURE framework.

The framework presented below produces results through the utilization of open‐source, modularized code packages with a process for evaluating fitness‐for‐purpose at both the data and method level. The code is publicly available for any organization to adopt and is flexible to run on any data source converted to the OMOP CDM [12]. As outlined in Figure 1, the framework has five main steps: Initiation, Database Diagnostics, Phenotype Alignment, Analytic Design and Implementation, and Results Interpretation.

#### 2.2.1 Initiation

##### 2.2.1.1 Signal Evaluation Request

The process is initiated when a request is made to include RWE in the evaluation of a safety signal as part of the PV signal management process. Safety signals can arise from internal or external sources including health authority requests, spontaneous reports, clinical trials, and peer‐reviewed literature.

##### 2.2.1.2 Translate Request

The first ASSURE step involves identification of the marketed medicinal product, which is designated as the exposure target, and identification of the outcome(s) of interest. Additional analytic design choices include the identification of relevant indication(s), potential candidate comparator(s), and time‐at‐risk. A request may result in multiple evaluations based on multiple comparators, indications, and/or outcome definitions. Pre‐computed baseline characteristics of exposure populations are used to empirically inform selection of a suitable comparator medical product [13]. The target, comparator(s), indication(s), and outcome(s) form the foundation of generating RWE to address a signal evaluation request. The time at risk, for example, the time relative to exposure start and end when the hypothetical elevated risk of an outcome could occur, is specified based on factors reported in the observed signal case time to onset and biological plausibility.

#### 2.2.2 Database Diagnostics

The second step utilizes a database diagnostic tool [14] to assess available data sources for the presence of the medication exposure target, comparator(s), indication(s), and outcome(s). Database diagnostics are performed across available databases to assess whether each database is fit‐for‐purpose for the signal evaluation query. Data sources which lack the required completeness of longitudinal follow‐up time will fail database diagnostics and be ineligible for further analysis. Database diagnostics comprise of precomputed metadata that can identify age‐based special populations such as infants and elderly. The tool does not replace expert knowledge of data provenance in determining suitability. The database diagnostic tool provides an estimated sample size for the target and produces output to assess whether the required data aspects for the indication(s) and outcome(s) are available. For example, if the safety evaluation outcome is myocardial infarction, which is most often expected to be observed during an inpatient (hospital) visit, the diagnostics check whether each database contains data from inpatient visits; a database passes only if it includes inpatient visits. Analyses fail diagnostics if the estimated target cohort sample size is less than 1000, there are no available outcome concepts, or a required source (e.g., inpatient visits) is unavailable across all available data sources.

#### 2.2.3 Phenotype Alignment

Johnson & Johnson maintains an internal library of phenotype definitions that have been developed for medicinal products, potential comparators, and indications, including many designated medical events (safety outcomes). Porta defined phenotype as “the observable properties, characteristics, or form of an organism or person produced by the genotype in synergy or interaction with the environment [15].” In RWE, we operate phenotypes as cohorts or sets of persons who satisfy specified criteria for defined durations of time. More precisely, an RWE phenotype is an operationalized, data‐driven definition of an observable and potentially time‐varying patient state, such as an exposure, indication, outcome, or covariate, constructed from observational health data using explicit algorithmic criteria [16, 17]. If a new phenotype is required, a clinical description of the condition of interest is created and translated utilizing a standardized phenotype development process [17]. In brief, this process involves a literature review to identify any previously published definitions. Identified phenotypes are replicated in the OMOP CDM. Further, the evaluation team supports the creation of additional candidate definitions as needed based on the clinical description of the outcome. We then used the Cohort Diagnostics tool [17] to characterize the existing and new candidate definitions and the PheValuator tool [18, 19] to estimate measurement error characteristics, that is, sensitivity, specificity, and positive and negative predictive value. After reviewing results, we either select one of the candidates as a new approved phenotype, revise one or more candidates until one is agreed by consensus, or decide that RWD are not suitable to capture the phenotype of interest. Analyses failed if the outcome cannot be translated into a computable phenotype with operating characteristics deemed sufficiently acceptable by the study team prior to analysis execution. Newly developed phenotypes are added to the internal phenotype library and may be reused or updated for future analyses.

#### 2.2.4 Analytic Design and Implementation

##### 2.2.4.1 Design Analysis

The fourth step is to design the analysis using the process steps outlined below. The request is translated into the inputs required by the standardized open‐source analytic R packages within the OHDSI HADES toolset, which are implemented for the ASSURE analysis design and execution [20]. Each estimation analysis requires the creation of multiple cohorts. Target and comparator exposure cohorts are specified with the required minimum duration of follow‐up time. Outcome cohorts were specified with an outcome clean window, which is the minimum time in days that can occur between two outcome events for a second event to be considered a new event, during which patients are typically excluded from the risk set. A time at risk period is also defined as the interval of time relative to the index target exposure at which the outcome event can occur. The time at risk period can be specified as while “on treatment” (often referred to as a per protocol in clinical trials) as specified by the cohort definition as the interval from exposure start to end or can be defined as a fixed time period after target medication initiation (such as 30 days post‐exposure start), which may include time after medication discontinuation. The code is flexible to allow for additional time at risk periods; for example, exposure grace periods can be added to the cohort definitions depending on the needs of the analysis.

The design requires the identification of negative control outcomes (NCOs) [21] to assess the validity of the results and to account for residual bias. NCOs are those believed not to be caused by the target medication or comparator; in the absence of residual bias effect size estimates for negative controls ideally should be close to the null. ASSURE typically employs a set of 50–100 NCOs to allow estimation of a systematic error distribution [22]. NCOs are selected by a semi‐automated process leveraging information from literature, product labels, and spontaneous reports to generate a list of candidate NCOs, followed by manual review [21].

Once an analytic specification is complete, a protocol is generated to document the analytic specifications in human readable form for the team to review and instantiate the analytic decisions prior to the conduct of any analysis. These standardized procedures, with defined inputs and outputs, create a reproducible specification that ensures consistent and transparent application of methods across quality checked data sources.

##### 2.2.4.2 Analysis

Depending on the analysis design choices, standardized sets of results are produced for characterization, patient‐level prediction, and population‐level causal effect estimation.

###### 2.2.4.2.1 Characterization

Characterization [20] provides descriptive statistics about the target, comparator, indication and outcome cohorts, including baseline features for the exposed populations and the incidence rate of the outcome within the target medication exposure population(s). The package also creates distribution plots showing the time between the target index and outcome dates, summarizes potential cases of medication de‐challenges and rechallenges, and baseline risk factors for the outcome by comparing the subgroups of the target medication population with and without the outcome of interest.

###### 2.2.4.2.2 Cohort Method (CM)

The comparative CM [20] compares the target medication population to a comparator population. Large‐scale propensity scores (LSPS) were used to adjust for potential confounding [23, 24]. LSPS includes all medications, diagnoses, procedures, demographics, and comorbidity indices observed before index, and fits the propensity score model using LASSO regularization. Although the software supports many types of propensity score adjustments, matching was used to minimize bias [25]. For results in a given database to be unblinded, analyses must pass a set of pre‐specified, standardized diagnostics [8]. Failure to meet pre‐specified thresholds prevents analyses in that database from being unblinded or from contributing to cross‐network meta‐analyses. Diagnostics are briefly described below.

- *Power*: The Minimum Detectable Relative Risk (MDRR) for a given observed sample size (after applying analytic exclusions) using an *α* = 0.05, *β* = 0.20 are calculated. The diagnostic failure threshold is an MDRR value of 10 or higher, which is used to blind individual database results. However, an individual database that only fails the MDRR diagnostic may still contribute to the meta‐analysis, since drawing inferences across multiple under‐powered analyses is still of interest.
- *Systematic error*: A systematic error distribution using the negative control estimates [26] is fit and summarized as the Expected Absolute Systematic Error (EASE) [8]. An EASE of 0 means all variation in negative control estimates can be explained by random error. The diagnostic failure threshold was prespecified prior to analysis and was typically an EASE value of 0.25 or higher.

For the comparative cohort design, the following additional diagnostics were used:

- *Equipoise*: A preference score, the linear transformation of the propensity score, is estimated. An equipoise measure is then computed as the proportion of the population with a preference score between 0.3 and 0.7 [27]. The diagnostic failure threshold is an equipoise value of less than 20%.
- *Covariate balance*: We compute the standardized mean difference (SMD) for every covariate used to balance any two exposure groups. The diagnostic fails when the absolute SMD of any covariate is 0.1 or higher [28].

Following evaluation of the diagnostics, the comparative CM evidence is synthesized, from analyses which pass pre‐specified diagnostics, with a meta‐analysis utilizing a Bayesian random‐effects model, to unblind the meta‐analysis the I [2] or the measure of between‐database heterogeneity in random‐effects models needed to be < 0.4 [29].

###### 2.2.4.2.3 Self‐Controlled Case Series (SCCS)

The SCCS design [20] defines time intervals within the same individual as exposed and unexposed periods based on the target and the time at risk. All time‐invariant potential confounders are inherently accounted for because each person serves as their own control. Within‐subject confounding can occur when the rate of the outcome and exposure change as a function of time. Time‐varying confounding due to calendar time and season is adjusted for using splines. For specific study designs dealing with children or the elderly, age can be used to adjust for time. For results to be unblinded, analyses must pass a set of pre‐determined, standardized diagnostics [8]. Each diagnostic has a failure threshold, which prevents unblinding of potentially biased results if not met. In addition to the MDRR and EASE diagnostics (Section 2.2.4.2.2), the SCCS employs two additional diagnostics, which are briefly described below.

- *Reverse causality*: A test if the risk of the outcome was already increased immediately prior to target exposure (30 days before versus 30 days after). The diagnostic failure threshold for reverse causality test is a *p*‐value < 0.01.
- *Time trend*: This diagnostic assesses whether the adjustment using splines is sufficient to control for time trends. Monthly expected outcome counts after spline adjustment are computed and compared to the observed counts. If the mean absolute observed‐to‐expected ratio significantly exceeds 1.25 with an alpha of 0.05 then this diagnostic fails.

Like the CM, following evaluation of the diagnostics the SCCS evidence is synthesized with a meta‐analysis utilizing a Bayesian random‐effects model; to unblind the meta‐analysis the I [2] or the measure of between‐database heterogeneity in random‐effects models needed to be < 0.4 [29, 30].

#### 2.2.5 Results Interpretation

##### 2.2.5.1 Review Objective Study Validity Diagnostics

Following execution across the databases that pass database diagnostics, the analysis results are stored in a results database. An interactive R Shiny application is used to explore the results. This application allows the research team to evaluate the characterization results, comparative CM and SCCS objective study validity diagnostics, and forest plots of unblinded analyses which passed study diagnostics for CM and SCCS results.

##### 2.2.5.2 Documentation

The final step is to review the analyses to document findings, including the comparative CM and SCCS results when available, and support safety decisions. If all estimation analyses fail across all databases, characterization results provide valuable documentation of outcome event rates within a target medication population and can help profile patients at higher risk of experiencing the outcome. These results are not used to construct observed over expected or unadjusted incidence rate ratios but can be used to contextualize the incidence rate across large RWD sources.

### 2.3 Evaluation of Framework

The framework aims to be (1) efficient, generating evidence within 60 days to inform regulatory responses, (2) applicable, addressing a wide range of signal evaluation requests and not producing evidence that is likely subject to potential bias in observational data, and (3) impactful, generating useful results that inform safety decisions.

To evaluate efficiency, we measured (1) the time between when the ASSURE team was notified of a signal evaluation request and when the results were generated, for target‐outcome combinations with any results, and (2) the time between when the ASSURE team was notified of a signal to evaluate and when the signal was determined infeasible, for target‐outcome combinations that failed database diagnostics or phenotyping.

Different types of diagnostics are included in the ASSURE framework to ensure the results are reliable. To gain insight into the applicability of observational data for signal evaluation, the main causes of each type of diagnostic failure were investigated.

Further investigation to evaluate impact included the number of signal evaluation requests where it was feasible to generate characterization results and estimation results (comparative cohort effect estimates and self‐controlled case series estimates). When analyses repeatedly failed to produce estimable results—defined as effect estimates with sufficient exposed cases, model convergence, and acceptable comparator propensity‐score overlap—we investigated whether the framework was unlikely to provide impactful RWE for signal evaluation, looking for causes such as low or absent exposure, low power based on incidence of the outcome, or poor propensity‐score balance.

## 3 Results

**FIGURE 2:**
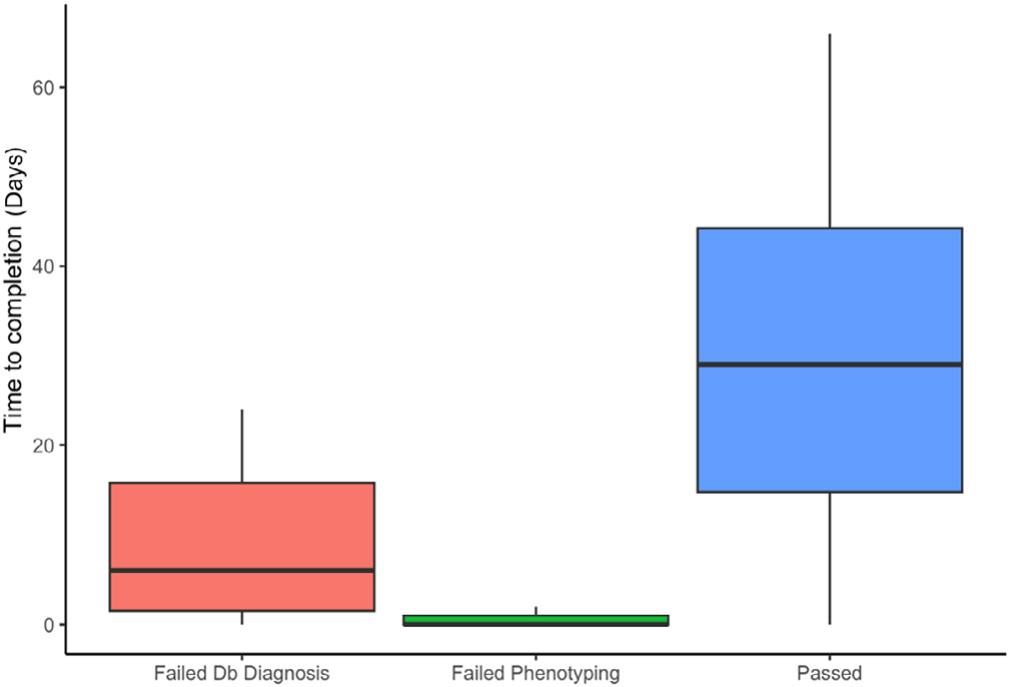
Time to completion in days stratified by the signal request output.

The ASSURE team received 47 signal evaluation requests that resulted in 110 unique target, comparator, indication, and outcome evaluations in the first 19 months of the program implementation. Forty‐five (96%) requests were completed within 60 days, with a median completion time of 23 days. The two requests that took longer than 60 days took 66 and 63 days. Figure 2 shows the time to complete requests in days stratified by output type (failed at database diagnostic, failed at phenotyping or passed). The median completion time was 2 days (max 24) for requests failing database diagnostics, 0 days (max 2) for requests failing at phenotyping, and 29 days (max 66) for requests where characterization and potentially estimation results were generated.

A total of 8 requests failed database diagnostics, all due to the target medication population having fewer than 1000 patients per database and 3 requests failed phenotyping due to inability to accurately identify any patients with the outcome. Eleven failed both comparative cohort and self‐controlled case series study diagnostics, resulting in only characterization results being generated. For the comparative CM, 7 of the 11 (64%) failed the MDRR diagnostics and 6 of the 11 (54%) failed shared covariate balance diagnostics. For the self‐controlled case series method, 6 of 8 (75%) failed the EASE statistic, 4 of the 8 (50%) failed the time‐trend diagnostic. A total of 22 requests failed study diagnostics to generate estimation results.

**FIGURE 3:**
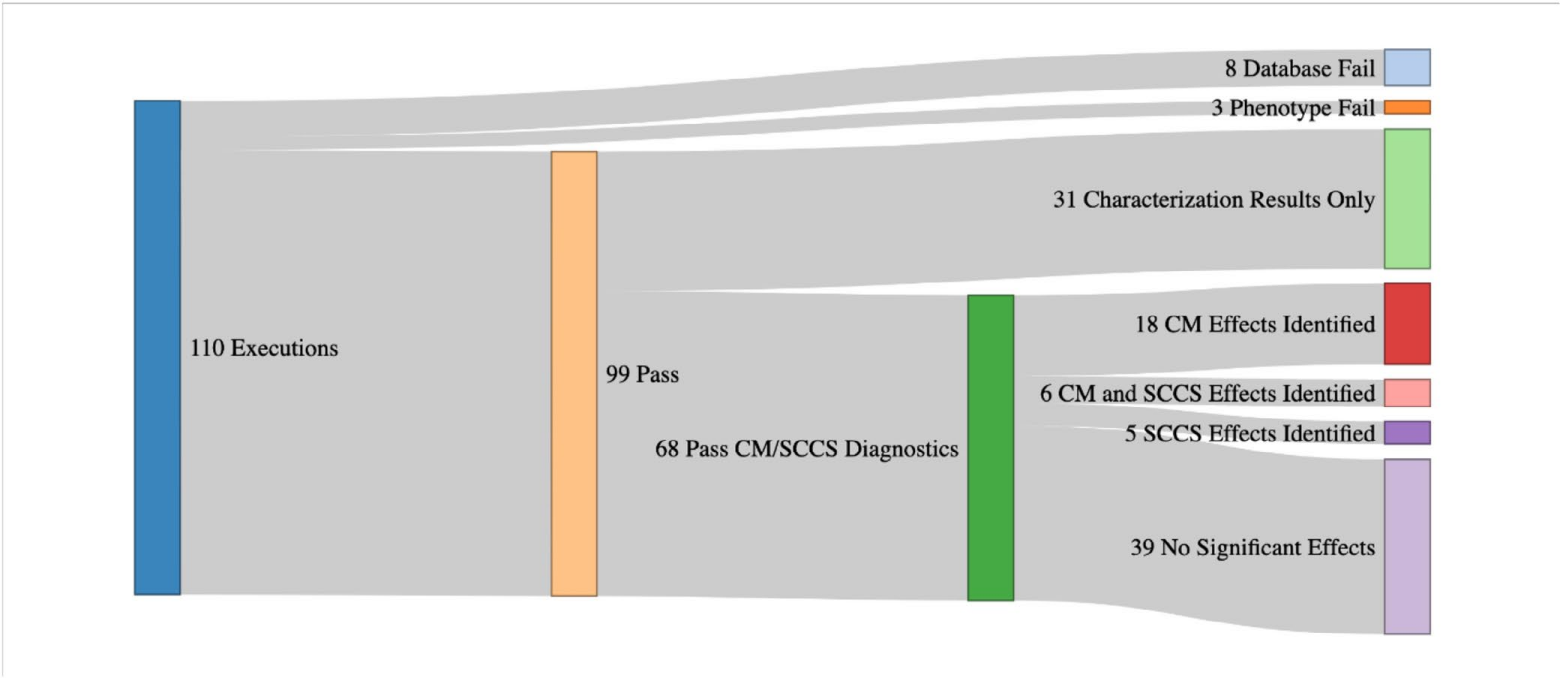
Output for each signal evaluation (target, comparator, indication and outcome). CM: Cohort method; SCCS: self‐controlled case series.

Figure 3 shows the disposition of requests and where requests and unique target, comparator, indication, and outcome evaluations failed database diagnostics, phenotype development, or objective study validity diagnostics. It was possible to generate at least some characterization results for 36 (77%) out of 47 signal evaluation requests, which corresponded to 99 (90%) of the total 110 executions. At least one estimation analysis was produced for 25 (53%) of the 47 signal evaluation requests, or 68 (62%) of the 110 executions. Of the 68 executions that passed analysis diagnostics, 21 passed both CM and SCCS diagnostics, 41 passed only CM diagnostics, and 6 passed only SCCS diagnostics. Figure 3 further breaks down the by method the effects identified. Supporting Information Figure S1 breaks down the results by signal request (target and outcome).

## 4 Discussion

The ASSURE framework provides rapid, transparent, and fit‐for‐purpose analytics applied to large RWD sources that generate RWE for safety signal evaluations. Implementation of the framework generates high‐quality evidence that can be integrated into routine safety decision making. This innovative approach at J&J utilizes the OMOP CDM and OHDSI analytic tools to address specific medication safety signal evaluation questions.

Implementation of ASSURE over a 19‐month period enabled us to generate RWE from observational healthcare data to aid signal evaluation for 77% of the requests, with nearly all requests being completed within 60 days (median 23 days). Reliable and informative estimation results from comparative CM or SCCS methods were produced for 54% of requests. The ability to provide timely and applicable RWE to inform most safety evaluations was feasible because of efficiency gained through the standardized data, standardized process, existing phenotype library, and use of open‐source software packages.

As a means of an example, one request asked the question “what is the risk of prostatitis following canagliflozin exposure in patients with type 2 diabetes mellitus?” This request was conducted across eight databases in less than 30 days. Canagliflozin initiators were compared with empagliflozin, dapagliflozin, sitagliptin, and liraglutide initiators. Meta‐analysis of results from all databases could not reject the null hypothesis of no association between canagliflozin and the prostatitis outcome compared to the comparator drug groups [31].

Most requests that failed to generate results were due to a database diagnostics requirement of a minimum of 1000 patients with the target medication exposure population. This lower observed exposure level occurred commonly for newly marketed medications given uptake trajectories and data lags in licensed RWD sources. In future work we recommend investigating whether this requirement could be relaxed, enabling more requests to generate characterization output even if estimation diagnostics still fail. When estimation diagnostics all failed, indicating a lack of power or some form of bias being present, we were still able to generate characterization results that provided incidence rates and described the medication user population (e.g., demographics, medical history and concurrent medication). Although these descriptive results do not provide causal insight, they were deemed useful by the teams evaluating the safety signals. We strongly recommend the blinding of potentially biased results from being reviewed and potentially misinterpreted in all observational research in secondary data sources.

Although we were able to generate CM and SCCS results for 54% of requests, the strength of evidence varied from one estimate in a single database to multiple estimates. across multiple databases enabling a meta‐analysis estimate. A meta‐analysis is performed when two or more databases produce effect estimates; meta‐analytic results are unblinded when the heterogeneity passed prespecified diagnostic thresholds. We considered whether there were statistically significant effects in only one database versus a significant effect in the meta‐analysis and whether results across databases were directionally consistent.

Despite the many strengths of the ASSURE framework, there are limitations. As with other observational studies using licensed health insurance and EHR data, source medical records cannot be obtained for this research; therefore, medical‐record–based validation of outcomes is not possible. While a standardized approach is utilized to generate computable phenotypes [17] some outcomes cannot be assessed using secondary RWD, leading to failures in phenotype diagnostics. Examples of outcomes that cannot be assessed using secondary RWD include conditions without a specific diagnosis code, such as pseudoaldosteronism. In addition, the ASSURE framework may fail database diagnostic and/or analysis diagnostics for newly marketed medications due to lower uptake and time lags in the RWD. Future work could investigate different methods that would enable reliable causal inference with smaller patient samples, related safety outcomes, or proxies for the analysis such as learning from medications in the same class [32]. However, diagnostic failures are an important measure to protect against generation of results that have the potential to be invalid. As with all RWD endeavors, the ability to measure the exposure of interest is critical; the framework cannot evaluate safety of devices given the challenges in identifying devices in RWD sources. Refinements to the framework are allowing for evaluation of in utero exposures, time‐varying exposures, and cumulative dose exposures as the code is flexible to expand into these areas. Finally, successful implementation of the ASSURE framework required a collaborative team with the right subject matter expertise in pharmacoepidemiology methods, clinical medicine, RWD assessment, and programming in collaboration with the broader cross‐functional safety management team. Success also depended upon access to a sufficient number of observational healthcare databases mapped to the OMOP CDM.

## 5 Conclusions

We proposed a standardized framework that generates applicable, transparent, and efficient RWE to support signal evaluation using observational healthcare data. Over a 19‐month period, we implemented ASSURE utilizing open‐source tools and demonstrated successful delivery of evidence for integration into most signal evaluations within short timeframes and encourage others to adopt this framework for RWE safety surveillance.

## Funding

This work was supported by Johnson & Johnson Innovative Medicine.

## Ethics Statement

ASSURE utilizes de‐identified administrative data and has been exempted from Institutional Review Board review.

## Conflicts of Interest

All authors are employees and shareholders of Johnson & Johnson Innovative Medicine.

## Supporting information


**Figure S1:** Output for each signal request on the request (target and outcome). CM: Cohort Method, SCCS: Self Controlled Case Series.

## Data Availability

The data that support the findings of this study are available from commercial data vendors. Restrictions apply to the availability of these data, which were used under license for this study. Data are available from the author(s) with the permission of commercial data vendors.
