# Supplementary material for: Active Safety Surveillance Using Real‐World Evidence (ASSURE) Implementation: Transparent and Reproducible Real‐World Evidence Standardized Framework to Support Safety Signal Evaluation
Source: Pharmacoepidemiol Drug Saf. 2026 Jul 24;35(8):e70435. doi: 10.1002/pds.70435 (PMC13397054; doi:10.1002/pds.70435)
Supplement: Supplementary file 1 — Figure S1: Output for each signal request on the request (target and outcome). CM: Cohort Method, SCCS: Self Controlled Case Series. [file PDS-35-e70435-s001.docx]

Supplemental Material


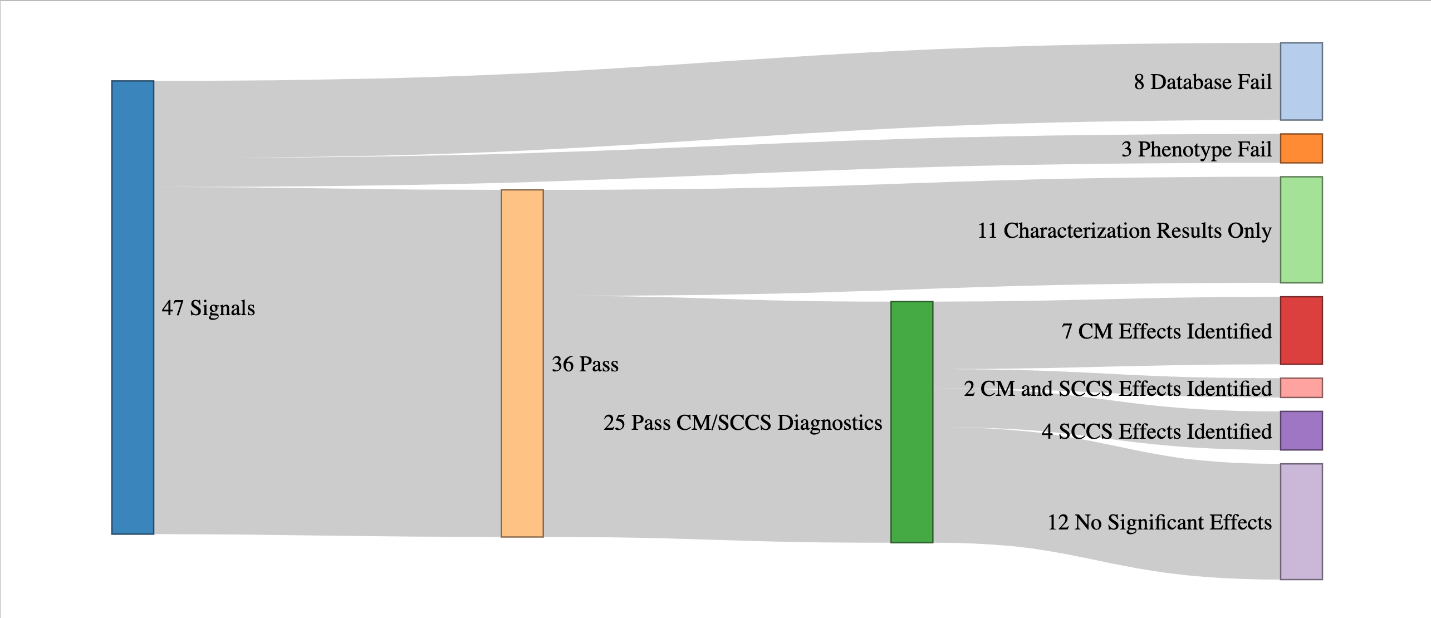


**Figure 4** –Output for each signal request on the request (target and outcome). CM: Cohort Method, SCCS: Self Controlled Case Series
